# Supplementary material for: Single-dose VSV-Sudan virus vaccine protects from lethal Sudan virus infection within one week: a challenge study in macaques: VSV-SUDV but not VSV-EBOV protects NHPs from Sudan virus disease
Source: bioRxiv. 2025 Mar 26:2025.03.26.645555. Preprint. [Version 1] doi: 10.1101/2025.03.26.645555 (PMC11974824; doi:10.1101/2025.03.26.645555)
Supplement: 1 [file NIHPP2025.03.26.645555V1-supplement-1.pdf]

bioRxiv preprint doi: https://doi.org/10.1101/2025.03.26.645555; this version posted March 26, 2025. The copyright holder for this preprint (which was not certified by peer review) is the author/funder. This article is a US Government work. It is not subject to copyright under 17 USC 105 and is also made available for use under a CC0 license.

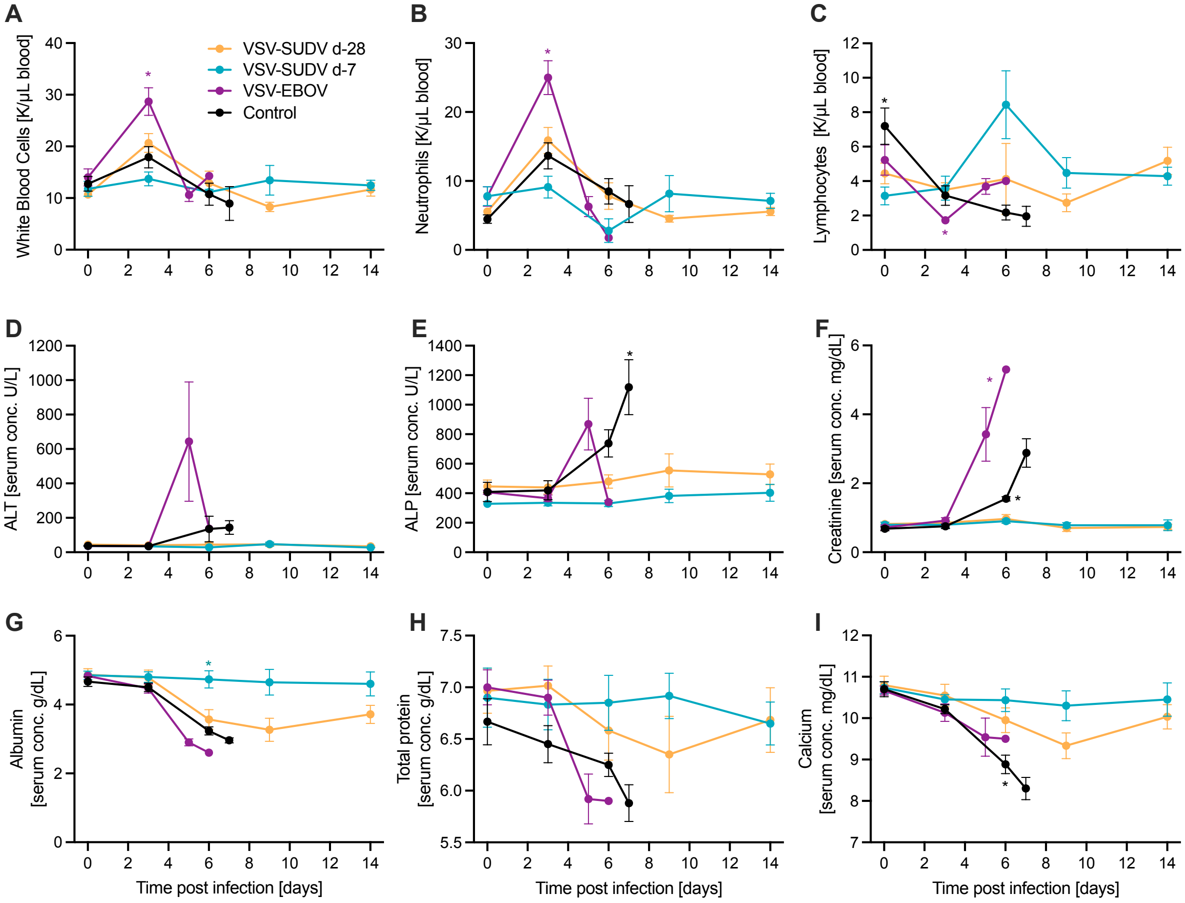

Figure S1. Changes in blood and serum parameters in NHPs after SUDV challenge. NHPs (n=6 per group) were vaccinated 28 days before challenge (d-28) with either VSV-SUDV, VSV-EBOV or control vaccine (VSV-LASV). Another group was vaccinated with VSV-SUDV 7 days before challenge (d-7). On day 0, all 24 NHPs were challenged with a lethal dose of SUDV. Changes in (A) white blood cell, (B) neutrophil, and (C) lymphocyte counts as well as serum levels of (D) alanine aminotransferase (ALT), (E) alanine phosphatase (ALP), (F) creatinine, (G) albumin, (H) total protein and (I) calcium are shown. Statistical significance was determined by two-way ANOVA with Tukey's multiple comparisons. Statistical significance is indicated as *p<0.05.

bioRxiv preprint doi: https://doi.org/10.1101/2025.03.26.645555; this version posted March 26, 2025. The copyright holder for this preprint (which was not certified by peer review) is the author/funder. This article is a US Government work. It is not subject to copyright under 17 USC 105 and is also made available for use under a CC0 license.

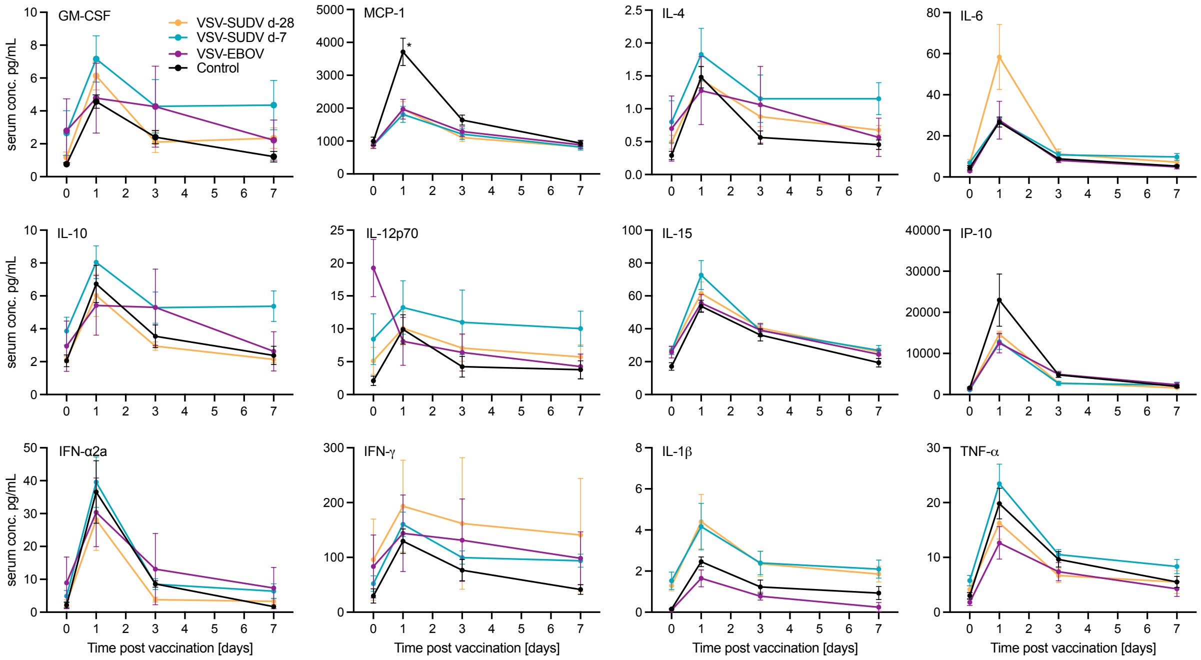

Figure S2. Levels of cytokines and chemokines in the serum after vaccination. Expression levels of selected cytokines or chemokines were determined after vaccination. Mean and standard error of the mean are depicted. Statistical significance was determined by two-way ANOVA with Tukey's multiple comparisons. Statistical significance is indicated as *$p<0.05$.

bioRxiv preprint doi: https://doi.org/10.1101/2025.03.26.645555; this version posted March 26, 2025. The copyright holder for this preprint (which was not certified by peer review) is the author/funder. This article is a US Government work. It is not subject to copyright under 17 USC 105 and is also made available for use under a CC0 license.

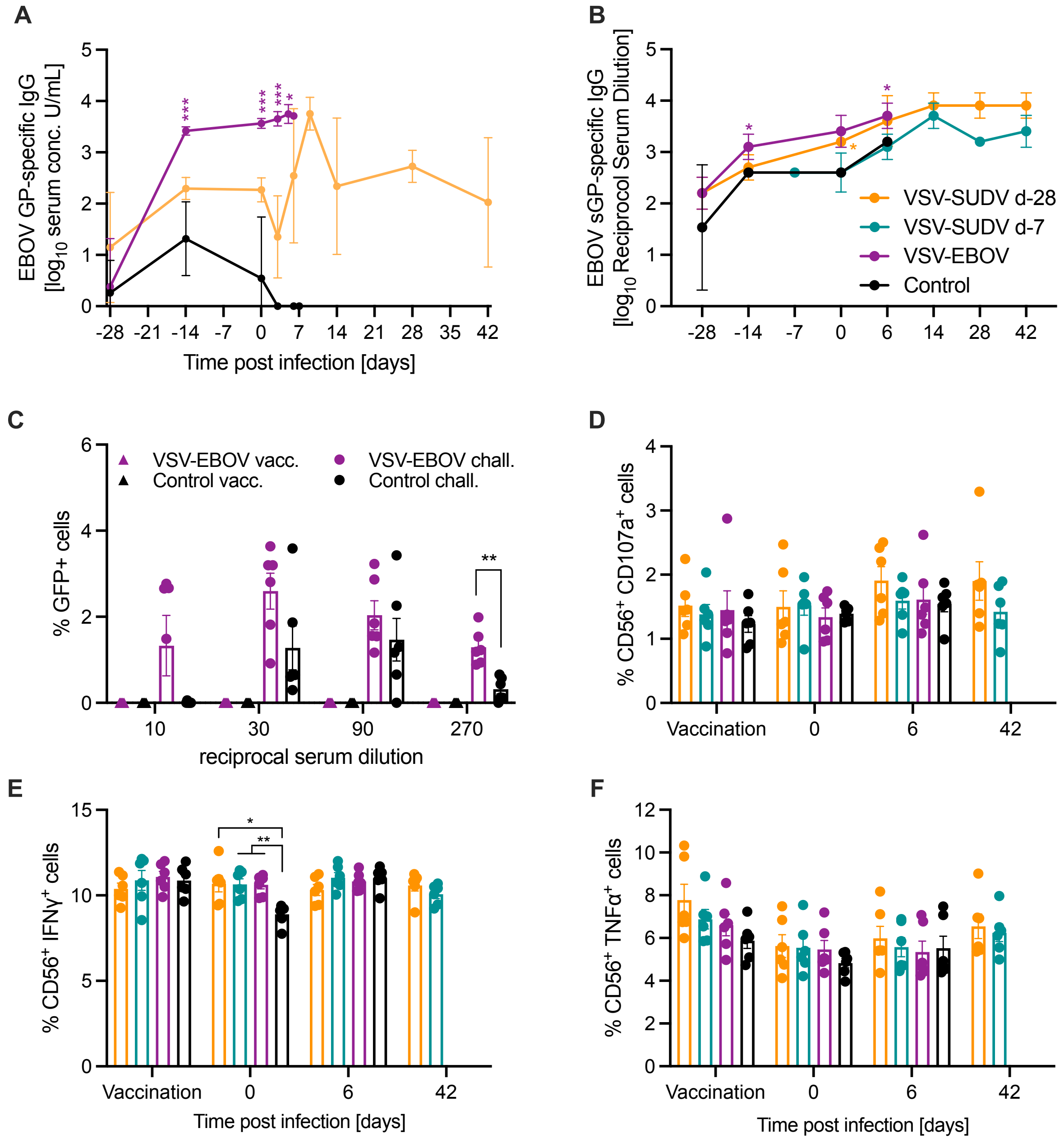

Figure S3. EBOV GP/sGP-specific IgG levels, antibody-dependent enhancement (ADE) and antibody-dependent killer cell activation (ADNKA). (A) EBOV GP-specific IgG responses in the serum of the 3 groups vaccinated 28 days before SUDV challenge. (B) EBOV sGP-specific IgG responses over time. (C) ADE on K562 cells with SUDV-GFP. ADNKA with (D) CD107^{+} cells, (E) IFNγ^{+} cells, and (F) TNFα^{+} cells. Geometric mean and geometric SD are depicted in A, B. Mean and standard error of the mean are shown in C-F. Statistical significance was determined by two-way ANOVA with Tukey's multiple comparisons (3 or 4 groups) or Sidak's multiple comparisons (2 groups). Statistical significance is indicated as **p*<0.05, ***p*<0.01, and ****p*<0.001.

bioRxiv preprint doi: https://doi.org/10.1101/2025.03.26.645555; this version posted March 26, 2025. The copyright holder for this preprint (which was not certified by peer review) is the author/funder. This article is a US Government work. It is not subject to copyright under 17 USC 105 and is also made available for use under a CC0 license.

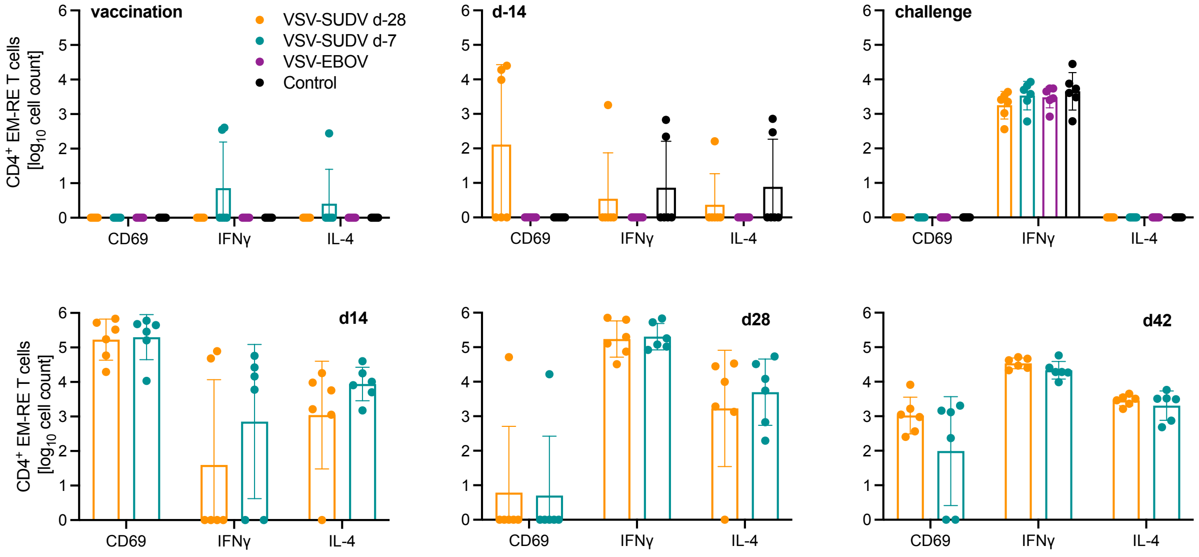

Figure S4. CD4 T cell responses after vaccination and challenge. PBMCs were stimulated with a SUDV GP-specific peptide pool and analyzed for CD4$^+$ effector memory re-expressing (EM-RE) T cells. Levels of CD69, IFNγ and IL-4 expression were analyzed throughout the study. Geometric mean and geometric SD are depicted. Data was analyzed by two-way ANOVA with Tukey's multiple comparisons and no statistical significance was determined between groups.

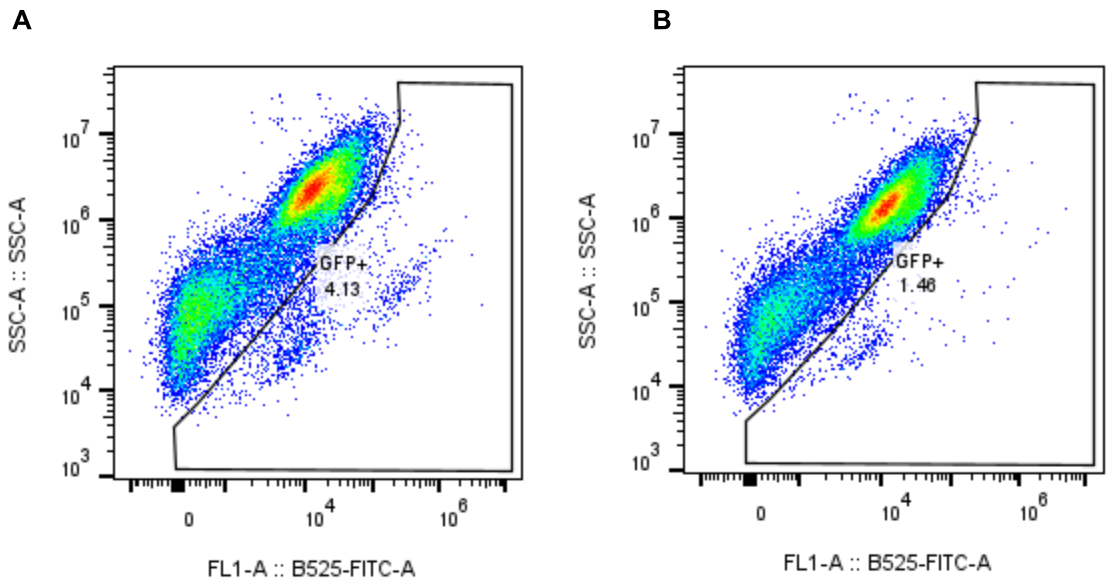

Figure S5. Gating strategy for neutralization and antibody-dependent enhancement (ADE) assays. Neutralization was assessed in Vero E6 cells with VSV-SUDV-GFP. ADE was assessed using K-562 cells and SUDV-GFP. GFP-positive cells were counted. Exemplary gating strategy for ADE with serum from a (A) VSV-EBOV-vaccinated NHP and (B) control NHP at the time of SUDV challenge.

bioRxiv preprint doi: https://doi.org/10.1101/2025.03.26.645555; this version posted March 26, 2025. The copyright holder for this preprint (which was not certified by peer review) is the author/funder. This article is a US Government work. It is not subject to copyright under 17 USC 105 and is also made available for use under a CC0 license.

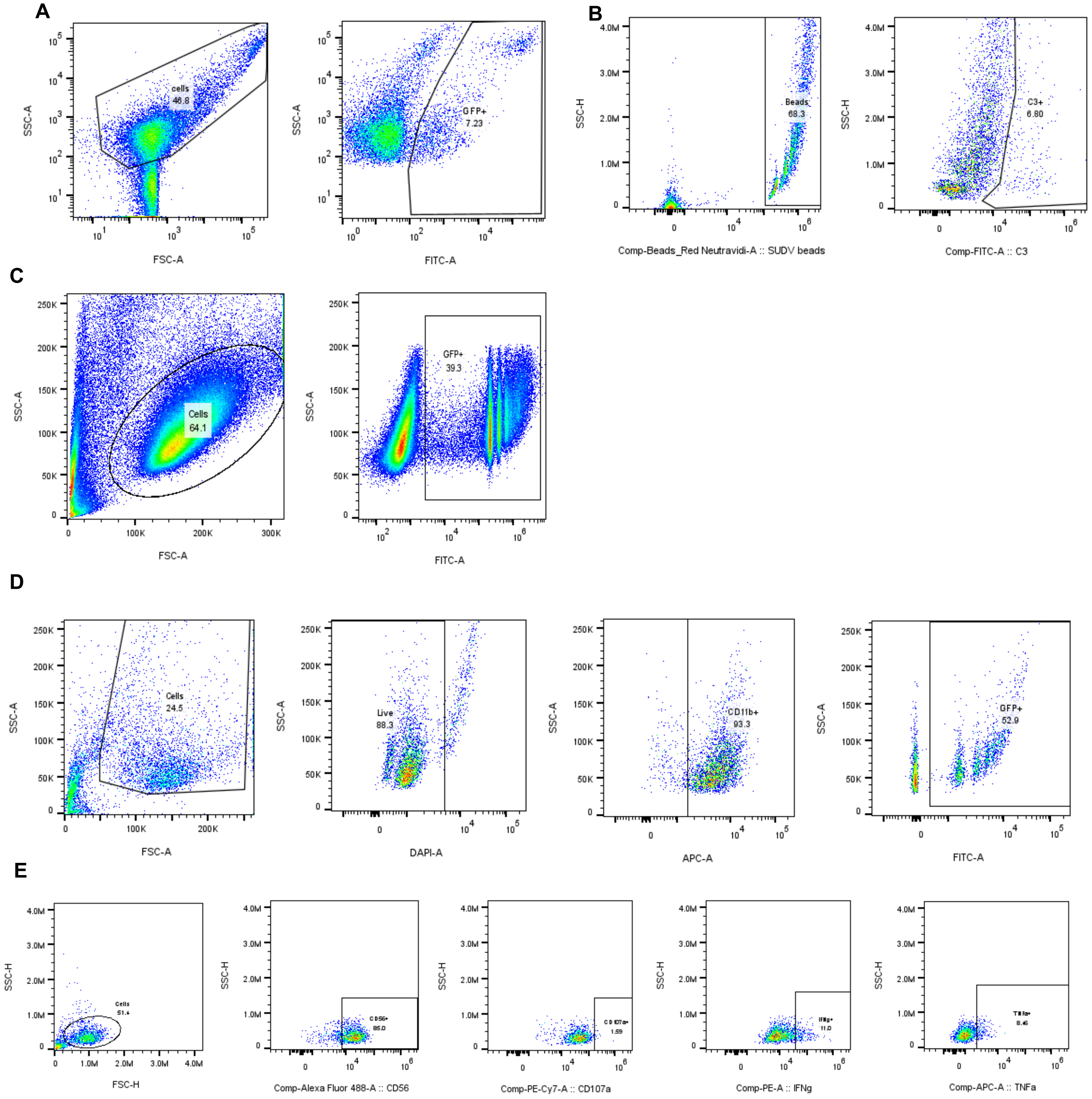

Figure S6. Gating strategy for antibody effector function analysis. (A) Neutralization. (B) Antibody-dependent complement deposition (ADCD). (C) Antibody-dependent cellular phagocytosis (ADCP). (D) Antibody-dependent neutrophil phagocytosis (ADNP). (E) Antibody-dependent natural killer cell activation (ADNKA).

bioRxiv preprint doi: https://doi.org/10.1101/2025.03.26.645555; this version posted March 26, 2025. The copyright holder for this preprint (which was not certified by peer review) is the author/funder. This article is a US Government work. It is not subject to copyright under 17 USC 105 and is also made available for use under a CC0 license.

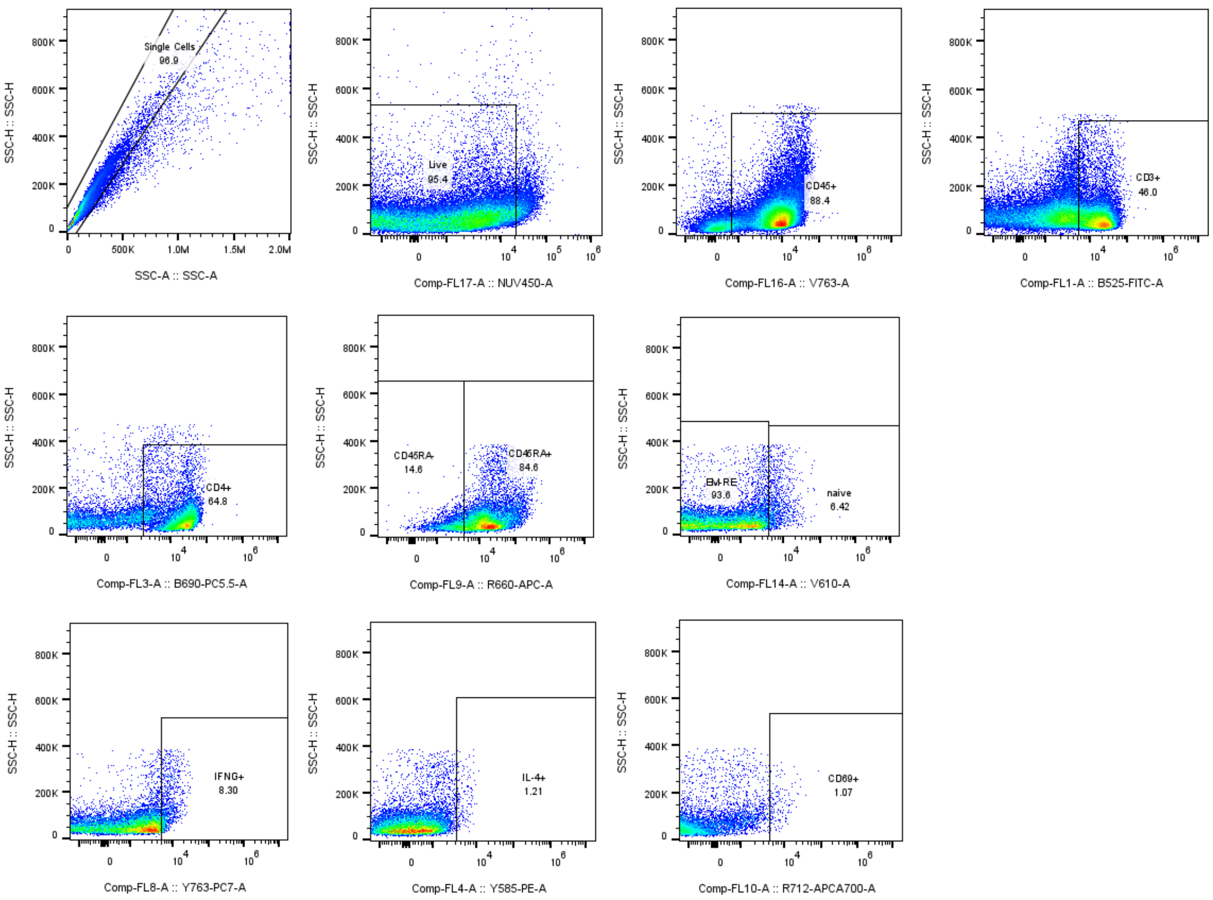

Figure S7. Gating strategy for functional analysis of vaccine-elicited T cells. Frozen PBMCs were thawed and restimulated overnight with a SUDV GP-specific peptide pool.
